# Breathing inequality: unmasking Liverpool’s air pollution burden on deprived youth

**DOI:** 10.1007/s10661-025-14594-2

**Published:** 2025-09-20

**Authors:** Jonathan E. Higham, Ian Sinha, Alice Lee, David Taylor Robinson, Olu Olajide, Sepeedeh Saleh

**Affiliations:** 1 Department of Geography and Planning, School of Environmental Sciences, University of Liverpool, Liverpool, UK; 2 Institute of Public Health, University of Liverpool, Liverpool, UK; 3 Alder Hey Children’s Hospital, Liverpool, UK

**Keywords:** Socioeconomic deprivation, Air pollution, Paediatric respiratory, Hospital admissions, Environmental justice

## Abstract

Liverpool, a city with an industrial legacy and among the most socioeconomically deprived local authorities in the UK, faces a significant health challenge: the combined impact of air pollution and deprivation on children’s respiratory health. This study deploys a dense network of 52 air quality sensors, one of the most comprehensive in the UK, to monitor particulate matter in 2023. PM2.5 levels ranged from 4.78 to 18.15 µg/m^3^ (median 7.15 µg/m^3^), and PM10 from 11.21 to 43.14 µg/m^3^ (median 17.30 µg/m^3^), frequently exceeding WHO thresholds. High concentrations were found in northern wards with high deprivation. Hospital admission rates for under-18 s ranged from 0.2 to 2%, exceeding national averages. Linear regression showed Index of Multiple Deprivation (IMD) scores explained 16.1% of the variance in hospital admissions (*R*^2^ = 0.1608, *β* = 0.023 to 0.025, *p* < 0.02), more than PM2.5 (6.6%) or PM10 (4.7%). Interaction terms suggested amplified pollution effects in deprived areas. Liverpool offers a valuable case study for understanding the intersection of environmental and social determinants of health as seen in many urban UK settings. Socioeconomic deprivation emerged as both a mediator, through factors like healthcare access, and a confounder in the pollution–health relationship. These findings underscore the need for targeted emission reductions and investment in disadvantaged communities. Future research with extended data could confirm these patterns and support broader policy action.

## Introduction

Air pollution represents a significant global public health challenge, exerting substantial morbidity and mortality impacts through its contribution to respiratory and cardiovascular diseases (Kampa & Castanas, 2008). In urban environments across the UK, this issue is exacerbated by anthropogenic emissions primarily from vehicular and industrial sources, with entrenched socioeconomic inequalities meaning vulnerable populations, including children, are disproportionately affected (Aithal et al., 2023; Hajat et al., 2015). Among these health effects, respiratory hospital admissions in children serve as a key indicator of environmental and social stressors.

Liverpool, a city with an industrial legacy and persistent socioeconomic challenges, provides a relevant context for analysing the intersection of air pollution and deprivation. Within the Liverpool City Region, air pollution is estimated to contribute to over 800 premature deaths annually, with the highest impact observed in socioeconomically deprived northern wards (Dajnak & Beevers, 2020). This burden is driven by traffic-related air pollution, a principal source of particulate matter, which has been closely linked to respiratory morbidity in children (Gehring et al., 2002; Khreis et al., 2018; Suhaimi et al., 2024). Hospital admission rates for asthma among children in Liverpool reached 228.4 per 100,000 in 2018–2019, exceeding the national average of 178.4 per 100,000, and highlighting significant health inequalities (Public Health Institute, 2020). These elevated rates reflect a broader trend in which urban, disadvantaged populations face increased exposure to PM2.5 and PM10, pollutants known to penetrate respiratory and cardiovascular systems, contributing to conditions such as asthma and pneumonia (Schraufnagel et al., 2019b), Through vulnerability to the effects of air pollution, socioeconomic disadvantage furthers environmental inequity (Institute of Medicine, 1999).

While extensive evidence has highlighted the adverse effects of particulate matter (PM), including PM2.5 and PM10, on respiratory health (Schraufnagel et al., 2019b), the nuanced relationships between socioeconomic deprivation and PM exposure as joint drivers of paediatric hospital admissions remain insufficiently explored. This gap in the literature is particularly important given the documented susceptibility of socioeconomically disadvantaged communities to elevated pollutant levels, compounded by restricted access to protective resources and healthcare (Hajat et al., 2015). Such inequities not only amplify individual health risks but also perpetuate broader inequities, underscoring the necessity for focused empirical investigation.

This study aims to address these gaps by systematically examining the associations among socioeconomic inequality, PM2.5 and PM10 exposure, and hospital admissions for respiratory conditions among children under 18 in Liverpool, UK. We focused on PM2.5 and PM10 due to their well-documented associations with respiratory morbidity in children, particularly asthma and pneumonia, which are prevalent in Liverpool (Schraufnagel et al., 2019a). While nitrogen dioxide (NO_2_) is a significant traffic-related pollutant, our sensor network was optimised for PM measurements, and PM sources (e.g., vehicular emissions, domestic combustion, and port activities) are prominent in Liverpool’s air quality profile (DEFRA, 2025). We prioritised respiratory hospitalisations over all-cause non-accidental admissions to capture outcomes most directly linked to air pollution exposure, given the established mechanistic pathways between PM and respiratory health (Kampa & Castanas, 2008). Future studies incorporating NO_2_ and broader health outcomes could complement these findings. Through an integrated spatial and statistical analysis of high-resolution air quality data, deprivation indices, and hospital admission records, this research seeks to deliver a robust quantitative evaluation of these interrelationships, thereby informing evidence-based environmental health policies and interventions. Specifically, this study addresses two research questions: (1) Are inequalities in PM2.5 and PM10 exposure across Liverpool associated with higher levels of socioeconomic deprivation? (2) To what extent do PM2.5, PM10, and deprivation, independently or jointly, influence respiratory hospital admissions among children?

## Methods

### Study setting

Liverpool, a major urban centre in northwest England, is characterised by a predominantly working-age population and a dynamic demographic profile, but is ranked the third most deprived local authority in England (Public Health Institute, 2020), with residents experiencing more than a quarter of their lives in poor health. Air pollution exacerbates these health disparities, contributing to increased rates of respiratory and cardiovascular morbidity. Major sources of air pollution include road traffic, which generates substantial emissions of nitrogen dioxide (NO₂) and particulate matter (PM), and industrial activity centred around the Mersey Estuary. The city’s legacy as a port and manufacturing hub contributes to longstanding air quality issues. Liverpool also has a history of public health leadership, from appointing the UK’s first Medical Officer of Health in the nineteenth century to ongoing collaborations between local authorities, the University of Liverpool, and NHS services, which have enabled the current analysis.

### Air quality monitoring

We deployed a network of 52 fixed-location air quality sensors, supplied by Aeternum and equipped with AlphaSense N Series Optical Particle Counters, across Liverpool in 2023. These sensors measured concentrations of PM2.5 (particles < 2.5 µm) and PM10 (particles 2.5–10 µm), along with temperature and humidity, at hourly intervals throughout the year. These sensors measured concentrations of PM2.5 (particles < 2.5 µm) and PM10 (particles 2.5–10 µm), along with temperature and humidity, at hourly intervals throughout the year. To ensure data accuracy, all sensors were calibrated against the DEFRA Automatic Urban and Rural Network (AURN) reference station in Speke, Liverpool, using a Golden node as an additional calibration standard. Calibration curves were applied to correct for meteorological influences, such as relative humidity and temperature, which can affect low-cost sensor performance. A machine learning model was employed to monitor sensor outputs continuously, detecting and correcting for baseline drift over the year-long monitoring period to maintain data reliability. Seasonal variation in solar gain affected the sampling frequency, with shorter intervals in summer and longer ones in winter. Sensors were strategically distributed, with higher density coverage in the city centre and densely populated areas. Annual mean values were calculated from these readings. Sensor data were aggregated by Lower Layer Super Output Areas (LSOAs) to align spatially with deprivation and hospital admission datasets.

### Deprivation and health data

Deprivation was assessed using the English Index of Multiple Deprivation (IMD) scores (2019), mapped to LSOA boundaries. Respiratory hospital admissions among individuals under 18 were obtained from the “Spirit Tree” hospital data system and normalised using 2021 ONS census data to calculate admissions as a percentage of the local under-18 population in each LSOA. This ensured comparability across areas with differing population sizes. Hospital admission was the primary outcome variable, with air pollution (PM2.5 and PM10) and deprivation (IMD) as key exposures.

### Statistical analysis

Our analysis followed a structured multi-step approach. First, we conducted descriptive analyses to visualise spatial distributions of air pollution, deprivation, and paediatric hospital admissions. We then used linear regression to examine the relationships between normalised respiratory admissions and the exposure variables (PM2.5, PM10, and IMD), modelled at the LSOA level.

For each pollutant, we constructed four models:

1. A univariate model with the pollutant as the sole predictor (PM2.5 Only or PM10 Only)
2. A univariate model with IMD score only (IMD Only)
3. A model incorporating both the pollutant and IMD (PM + IMD)
4. An interaction model including the pollutant, IMD, and their product term (PM × IMD)

Model performance was evaluated using *R*^2^ and adjusted *R*^2^ values, as well as the Akaike information criterion (AIC) and Bayesian information criterion (BIC), with lower values indicating better fit. Regression coefficients (*β*) and *p*-values were reported for all predictors. To ensure the validity of linear regression assumptions, we assessed the distributions of PM2.5 and PM10 concentrations for skewness. Both pollutants exhibited right-skewed distributions, as expected given the range and median values (PM2.5 4.78–18.15 µg/m^3^, median 7.15 µg/m^3^; PM10 11.21–43.14 µg/m^3^, median 17.30 µg/m^3^). We explored logarithmic transformations of PM2.5 and PM10 data to address skewness. However, regression models using transformed data did not significantly improve model fit (based on *R*^2^ and AIC) compared to untransformed data, and untransformed models met residual normality assumptions adequately, as confirmed by Shapiro–Wilk tests (*p* > 0.05). Thus, we retained untransformed PM data for interpretability and consistency with prior studies. We used *F*-tests to compare additive and interaction models, assessing whether the interaction terms improved model fit.

## Results

**Fig. 1:**
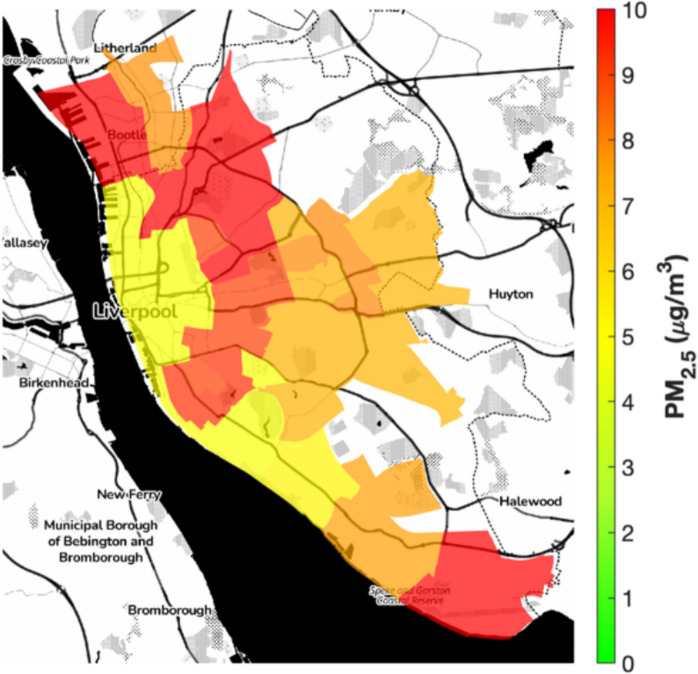
Annual mean PM2.5 concentrations (µg/m^3^) across Liverpool City Region wards in 2023, highlighting urban hotspots in red/orange

**Fig. 2:**
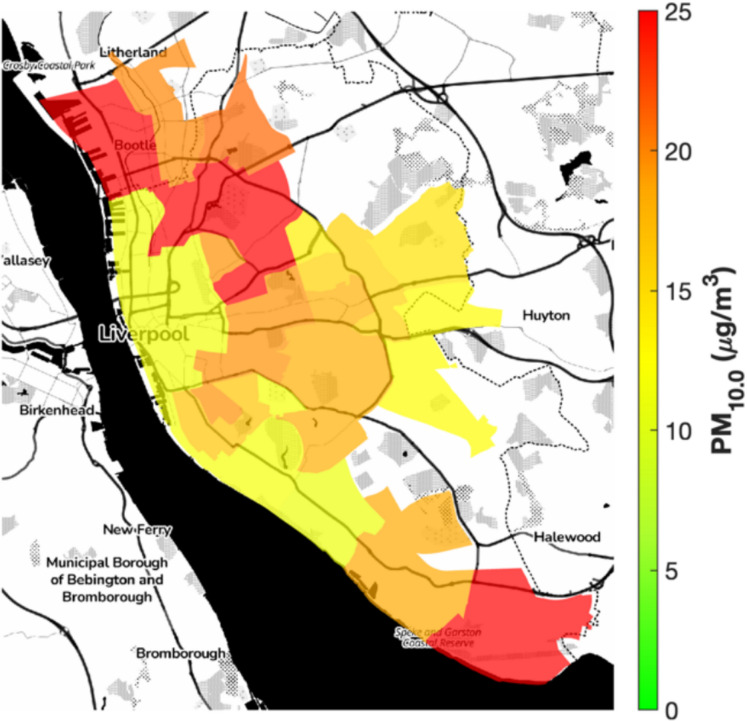
Annual mean PM10 concentrations (µg/m^3^) across Liverpool in 2023, showing elevated levels in densely populated areas

Ambient air quality assessments in Liverpool for 2023 revealed widespread exceedances of health thresholds. Annual mean PM2.5 concentrations ranged from 4.78 to 18.15 µg/m^3^ (median 7.15 µg/m^3^), surpassing the World Health Organization’s guideline of 5 µg/m^3^ across most wards (World Health Organization, 2021). These values align closely with data from the DEFRA AURN reference station in Speke, which reported an annual mean PM2.5 concentration of 7.02 µg/m^3^ in 2023, validating the reliability of Our sensor network. Temporal coverage was near-continuous, with sensors recording hourly data across 98% of the year, with minor gaps due to maintenance or power issue. Peak levels, reaching 18.15 µg/m^3^, were observed in the urban core and northern wards, driven by vehicular traffic and industrial activity (DEFRA, 2025). Figure 1 illustrates the distribution of PM2.5 across the region, showing high levels in dense urban and residential neighbourhoods, where high building density and limited vegetation constrain pollutant dispersion (Kumar, (Kumar n.d.)). PM10 concentrations ranged from 11.21 to 43.14 µg/m^3^ (median: 17.30 µg/m^3^), also frequently exceeding WHO thresholds (15 µg/m^3^) (World Health Organization, 2021). Geographical patterns for PM10 are shown in Fig. 2, which again highlights elevated levels in densely populated areas, largely attributable to vehicular emissions and domestic combustion sources.

**Fig. 3:**
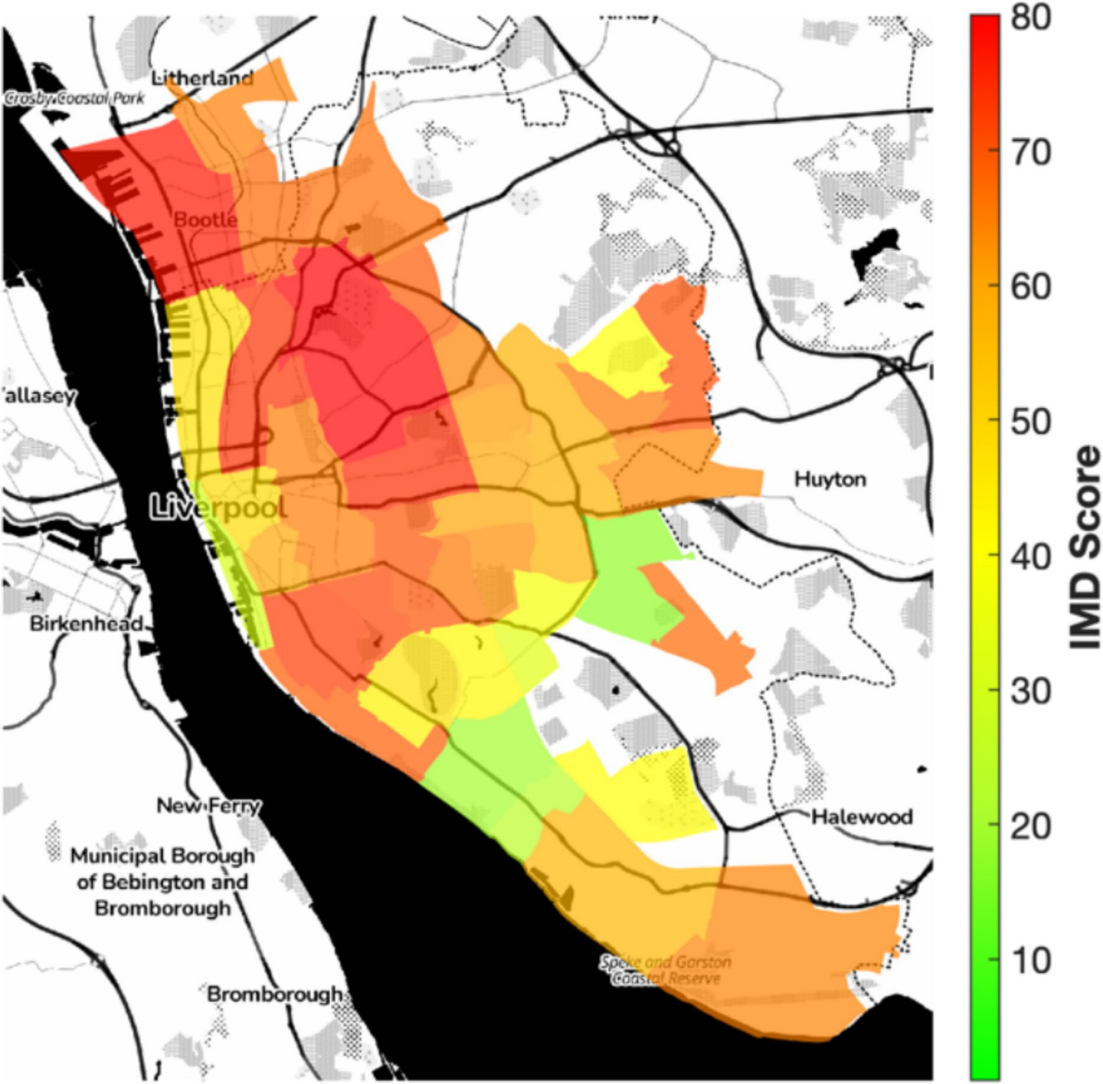
Index of multiple deprivation (IMD) deciles by LSOA in Liverpool (2019), with red/orange indicating the most deprived 50% nationally

**Fig. 4:**
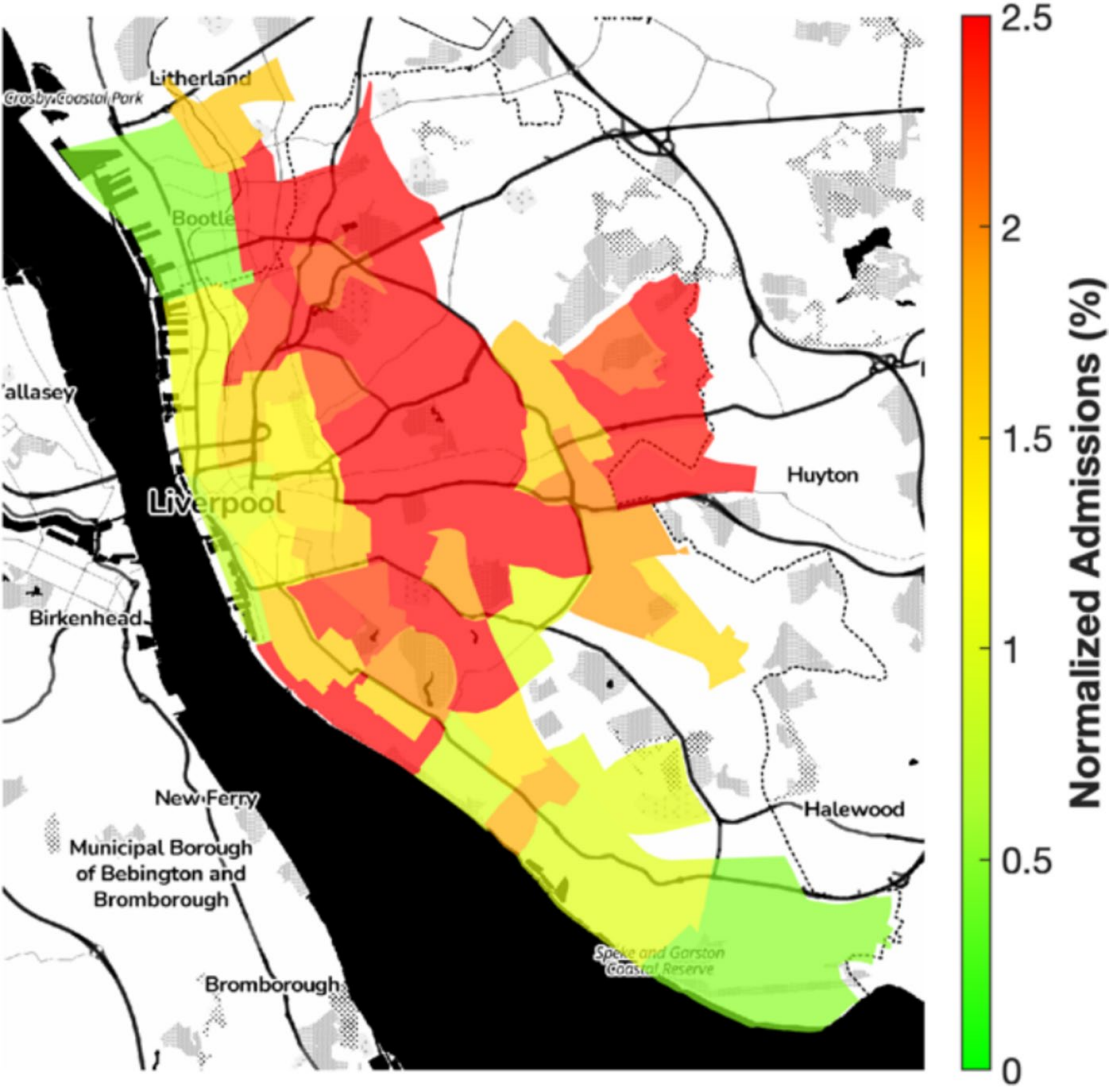
Spatial distribution of emergency hospital admissions for respiratory conditions among children under 18 in 2023, expressed as a percentage of LSOA under-18 populations (ONS 2021 census)

Over half of Liverpool’s Lower Layer Super Output Areas (LSOAs) rank among the most deprived nationally, with patterns of deprivation closely mirroring those of elevated air pollution, particularly in northern wards (Fig. 3) (Dajnak & Beevers, 2020). Hospital admission rates for respiratory conditions among children under 18 ranged from 0.2% to approximately 2% of the under-18 population per LSOA (ONS 2021 census), equivalent to 200 to 2000 per 100,000, exceeding the national average of 178.4 per 100,000 (Public Health Institute, 2020). These higher rates were concentrated in the same northern areas with the highest PM concentrations and deprivation scores (Fig. 4), suggesting overlapping spatial risk.

**Fig. 5:**
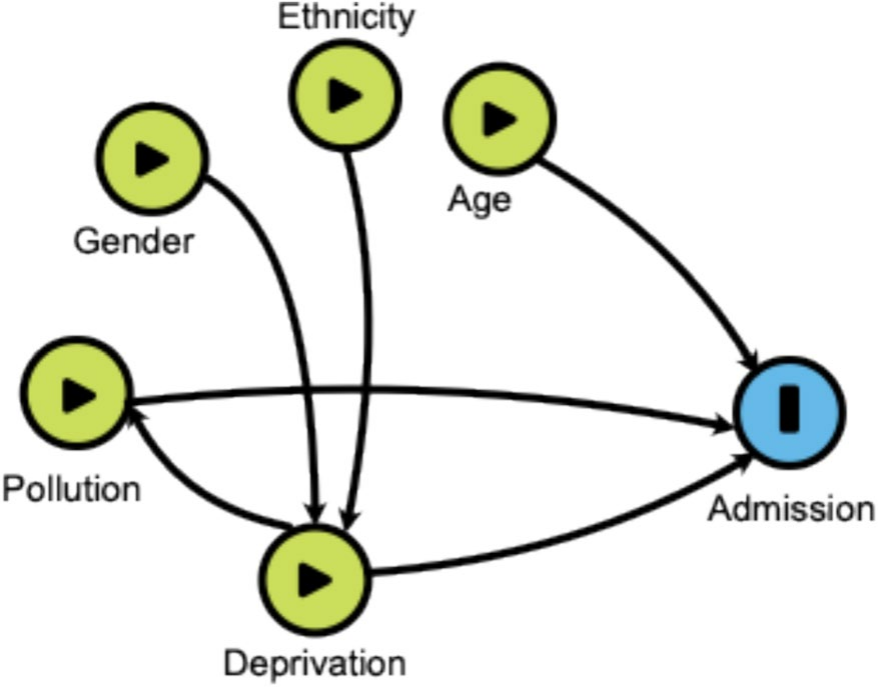
Directed acyclic graph depicting interactions between deprivation, air pollution, and paediatric respiratory admissions, with mediating and confounding pathways

To elucidate the hypothesised causal pathways, we developed a directed acyclic graph (DAG) to represent relationships among deprivation, air pollution, and paediatric respiratory admissions. In the DAG, arrows indicate hypothesised causal directions: deprivation influences both pollution exposure (via residential proximity to emission sources) and admissions (via healthcare access and susceptibility factors). Demographic factors, including age, gender, and ethnicity, were included as potential confounders affecting exposure and admissions. Specifically, age influences admissions due to varying respiratory susceptibility across childhood stages but does not directly affect pollution levels, as pollution is determined by environmental factors. Gender and ethnicity were modelled as influencing deprivation (e.g., through socioeconomic disparities) and exposure (e.g., via behavioural or residential patterns), but not age, as age is an independent demographic characteristic. To enhance clarity, we separated gender and ethnicity in the DAG to reflect their distinct roles in exposure and health outcomes. To guide our modelling approach and avoid inappropriate statistical adjustments, we developed a directed acyclic graph (DAG) to represent hypothesised causal relationships among key variables (Fig. 5). The DAG reflects the assumption that deprivation influences both air pollution exposure and paediatric respiratory health, positioning deprivation as a confounder in the relationship between pollution and hospital admissions. Conversely, if the primary focus is on the effects of deprivation, air pollution may act as a mediator. This framework informed decisions about which variables to adjust for in each model and how to interpret the results in relation to confounding and mediation.

**Fig. 6:**
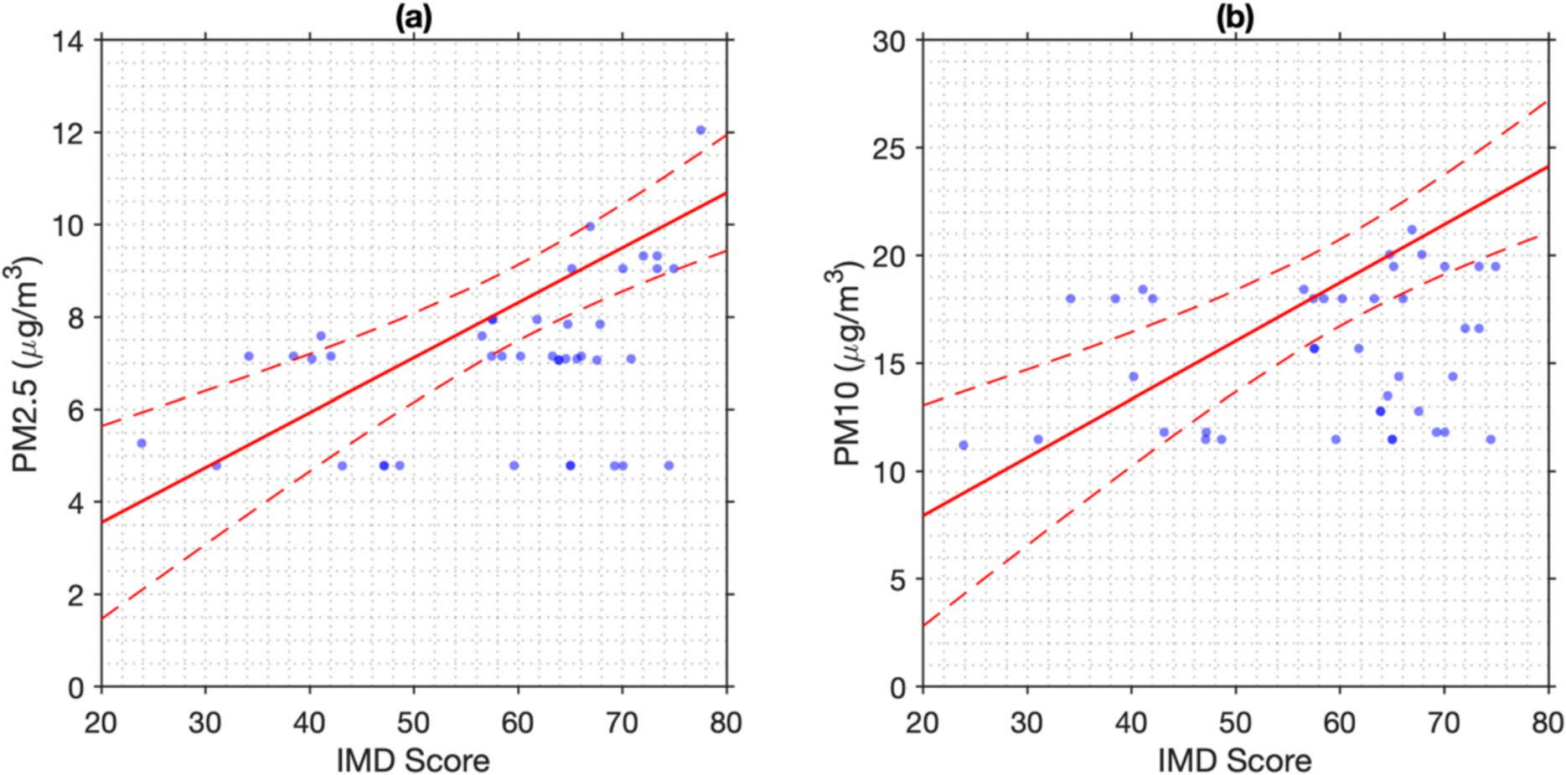
Relationship between IMD and particulate matter concentrations. **a** Scatter plot of PM2.5 (μg/m^3^) versus IMD with linear regression line (red) and 95% confidence intervals (red dashed lines). **b** Scatter plot of PM10 (μg/m^3^) versus IMD with linear regression line (red) and 95% confidence intervals (red dashed lines). Data points represent ward-level measurements

Linear regression was used to explore the relationship between IMD and ward-level PM2.5 and PM10 concentrations (μg/m^3^), which are summarised in Table 1. Figure 6 displays side-by-side scatter plots: (a) PM2.5 and (b) PM10 versus IMD (20–80 range), each with a regression line and 95% confidence intervals, showing a positive association between deprivation and pollution. Table 1 summarises the regression statistics. For PM2.5, *R*^2^ = 0.324 (adjusted *R*^2^ = 0.311) indicates 32.4% of variance is explained by IMD, with a significant slope of 0.119 μg/m^3^ per unit (*p* = 7.08 × 10^−6^, SE = 0.024), suggesting a 7.14 μg/m^3^ increase over the 60-unit range. PM10 has *R*^2^ = 0.291 (adjusted *R*^2^ = 0.278), with a steeper slope of 0.270 μg/m^3^ (*p* = 2.53 × 10^−5^, SE = 0.058), implying a 16.20 μg/m^3^ rise. AIC and BIC values (274.07 and 278.05 for PM2.5; 370.89 and 374.87 for PM10) reflect model fit. These findings confirm higher IMD values correlate with elevated PM levels, with PM10 showing a stronger link. The significant slopes highlight potential environmental justice issues, though moderate *R*^2^ values suggest other factors also influence air quality.

**Table 1:** Linear regression statistics for the relationship between IMD and particulate matter concentrations. The table presents *R*^2^, adjusted *R*^2^, Akaike information criterion (AIC), Bayesian information criterion (BIC), slope coefficient, standard error of the slope (slope SE), and *p*-value of the slope (slope *p*-value) for PM2.5 and PM10 versus IMD. Models are based on ward-level data

Metric	PM2.5 vs IMD	PM10 vs IMD
*R*^2^	0.32397	0.29131
Adjusted *R*^2^	0.31097	0.27768
AIC	274.07	370.89
BIC	278.05	374.87
Slope	0.11892	0.26994
Slope SE	0.023822	0.058386
Slope *p*-value	7.0828e − 06	2.5336e − 05

**Fig. 7:**
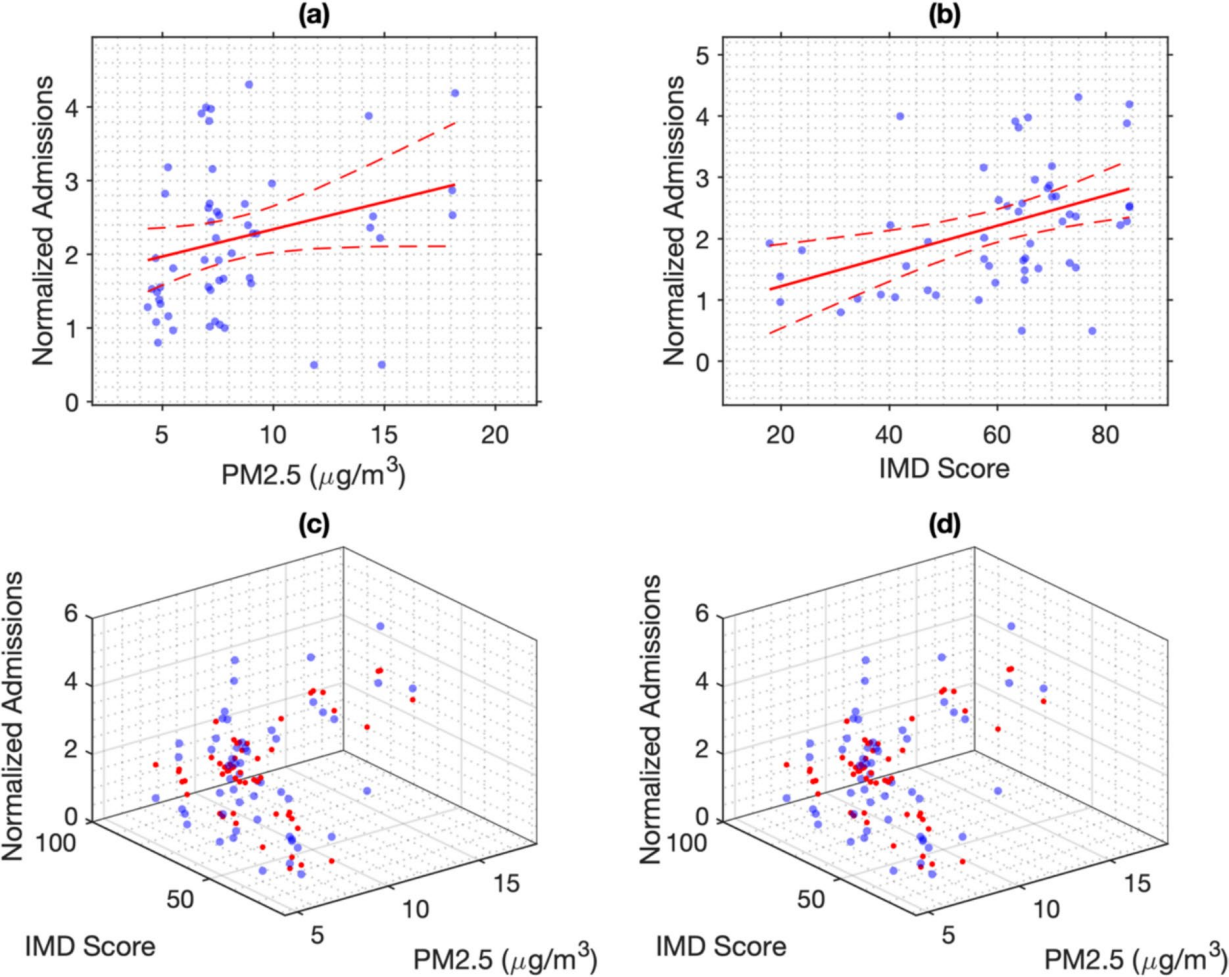
Relationships between PM2.5, IMD, and paediatric respiratory admissions across Liverpool wards. **a** Normalised admissions plotted against PM2.5 concentrations with linear regression fit and 95% confidence intervals (Model 1: PM2.5 only). **b** Normalised admissions plotted against IMD with linear regression fit and 95% confidence intervals (Model 2: IMD only). **c** 3D scatterplot showing observed admissions alongside predicted values from an additive linear model including both PM2.5 and IMD as independent predictors (Model 3: PM2.5 + IMD). **d** 3D scatterplot showing observed admissions and predicted values from a model including an interaction term between PM2.5 and IMD (Model 4: PM2.5 × IMD), allowing the effect of pollution to vary across levels of deprivation

To assess the statistical relationships between pollution, deprivation, and admissions, we applied linear regression models using standardised LSOA-level data (Tables 2–3; Fig. 7). For PM2.5, the IMD only model showed the best overall fit, explaining 16.1% of the variance in normalised paediatric admissions (*R*^2^ = 0.1608, adjusted *R*^2^ = 0.1446, AIC = 153.79, BIC = 157.77). The PM2.5 only model explained 6.6% (*R*^2^ = 0.0657, adjusted *R*^2^ = 0.0478), with poorer fit (AIC = 159.58). The additive PM2.5 + IMD model slightly improved explanatory power (*R*^2^ = 0.1618), but the interaction model (PM2.5 × IMD) did not meaningfully enhance fit, and its increased complexity was penalised (adjusted *R*^2^ = 0.1115, AIC = 157.72, BIC = 165.68).

**Table 2:** Model fit statistics for regression models predicting normalised paediatric respiratory admissions using PM2.5 and IMD in Liverpool wards. Models include PM2.5 alone (PM2.5 only), IMD alone (IMD only), additive effects (PM2.5 + IMD), and interaction effects (PM2.5 × IMD). *R*^2^, adjusted *R*^2^, AIC, and BIC are reported

Model	*R*^2^	Adjusted *R*^2^	AIC	BIC
PM2.5 only	0.0657	0.0478	159.58	163.56
IMD only	0.1608	0.1446	153.79	157.77
PM2.5 + IMD	0.1618	0.1289	155.72	161.69
PM2.5 × IMD	0.1618	0.1115	157.72	165.68

**Table 3:** Model fit statistics for regression models predicting normalised paediatric respiratory admissions using PM10 and IMD in Liverpool wards. Models include PM10 alone (PM10 only), IMD alone (IMD only), additive effects (PM10 + IMD), and interaction effects (PM10 × IMD). *R*^2^, adjusted *R*^2^, AIC, and BIC are reported

Model	*R*^2^	Adjusted *R*^2^	AIC	BIC
PM10 only	0.0467	0.0283	160.68	164.65
IMD only	0.1608	0.1446	153.79	157.77
PM10 + IMD	0.1608	0.1278	155.79	161.76
PM10 × IMD	0.1630	0.1127	157.65	165.61

A similar pattern was observed for PM10. The IMD only model again provided the strongest fit (*R*^2^ = 0.1608, adjusted *R*^2^ = 0.1446, AIC = 153.79), followed by the additive PM10 + IMD model (*R*^2^ = 0.1608, adjusted *R*^2^ = 0.1278, AIC = 155.79). The PM10 only model explained just 4.7% of variance (*R*^2^ = 0.0467), and the interaction model (PM10 × IMD) did not significantly improve fit (*R*^2^ = 0.1630, adjusted *R*^2^ = 0.1127), with higher AIC and BIC values indicating overfitting.

Regression coefficients for PM2.5-related models are presented in Table 4. In the univariate PM2.5 only model, PM2.5 was positively associated with respiratory admissions (*β* = 0.0759), but the effect did not reach conventional statistical significance (*p* = 0.0613). This suggests a potential increase in hospitalisation rates with higher PM2.5 exposure, although the evidence was marginal. When both PM2.5 and IMD were included (PM2.5 + IMD model), the effect of PM2.5 attenuated substantially (*β* = 0.0118, *p* = 0.8006), while IMD remained significant (*β* = 0.0233, *p* = 0.0192), indicating that deprivation had an independent and stronger relationship with admission rates. The interaction model (PM2.5 × IMD) introduced a three-term structure but yielded a non-significant interaction coefficient (*p* = 0.9778), with no improvement in overall fit, as confirmed by an *F*-test (*p* = 0.9777).

**Table 4:** Regression coefficients, standard errors, and *p*-values for models predicting normalised paediatric respiratory admissions with PM2.5 and IMD in Liverpool wards. Model 1 includes PM2.5 only, Model 3 includes PM2.5 and IMD additively, and Model 4 includes their interaction

Model	Term	Estimate	Standard error	*p*-value
Model 1	PM2.5	0.0759	0.0397	0.0613
Model 2	IMD	0.0246	0.0078	0.0026
Model 3	PM2.5	0.0118	0.0463	0.8006
Model 3	IMD	0.0233	0.0096	0.0192
Model 4	PM2.5	0.0187	0.2526	0.9413
Model 4	IMD	0.0239	0.0232	0.3090
Model 4	PM2.5:IMD	−0.0001	0.0033	0.9778

Table 5 displays the regression coefficients for models incorporating PM10. In the unadjusted PM10 only model, PM10 was positively associated with admissions (*β* = 0.0267), but again not significantly (*p* = 0.1167). When adjusted for deprivation (PM10 + IMD model), the PM10 effect effectively disappeared (*β* =  − 0.0001, *p* = 0.9948), while the IMD effect remained statistically significant (*β* = 0.0247, *p* = 0.0112). As with PM2.5, the interaction term in the PM10 × IMD model was not significant (*p* = 0.7182), and the *F*-test confirmed that the interaction did not contribute meaningfully to model fit (*p* = 0.7896).

**Table 5:** Regression coefficients, standard errors, and p-values for models predicting normalised paediatric respiratory admissions with PM10 and IMD in Liverpool wards. Model 1 includes PM10 only, Model 3 includes PM10 and IMD additively, and Model 4 includes their interaction

Model	Term	Estimate	Standard error	*p*-value
Model 1	PM10	0.0267	0.0167	0.1167
Model 2	IMD	0.0246	0.0078	0.0027
Model 3	PM10	−0.0001	0.0188	0.9948
Model 3	IMD	0.0247	0.0094	0.0112
Model 4	PM10	−0.0384	0.1071	0.7216
Model 4	IMD	0.0176	0.0218	0.4221
Model 4	PM10:IMD	0.0005	0.0014	0.7182

Taken together, these results suggest that while air pollution, particularly PM2.5, shows some association with paediatric respiratory admissions, socioeconomic deprivation is a more consistent and statistically robust predictor. Assessing relationships between the three terms suggests that the effects of deprivation on the health outcome in question also incorporate pollution effects, with no statistically detectable impact of pollution occurring independently of deprivation effects in this analysis. The spatial overlap between high PM concentrations, high deprivation, and high admission rates and complex interactions between the factors indicates an important area for further study.

## Discussion

Our data show that much of Liverpool—a region with significantly higher deprivation than national averages—is exposed to particulate matter concentrations above WHO annual thresholds for both PM2.5 and PM10 (World Health Organization, 2021). High levels of air pollution appear to be driven by traffic emissions and domestic sources, with the density of buildings further trapping pollution particles (DEFRA, 2025; Kumar, (Kumar n.d.)). In contrast, more affluent, sparsely populated areas tend to experience lower pollution levels due to reduced emissions and increased vegetation, with greenspace improving air flow and dispersion (Kumar, (Kumar n.d.)). Our analysis confirmed the correlation between deprivation and pollution exposure, highlighting multiple interacting risks to health and wellbeing. Inequalities in air pollution exposures have been widely reported in both UK and international studies (Boing et al., 2022; Fairburn et al., 2019; Fecht et al., 2015), although comparisons across rural and urban settings, and between Eastern and Western Europe, introduce important nuances (Milojevic et al., 2017; Richardson et al., 2013). In assessing the relationships between PM, deprivation, and paediatric respiratory admissions using linear regression (illustrated in Fig. 5), deprivation emerged as the strongest predictor. IMD alone explained 16.1% of the variation in admissions, compared to 6.6% for PM2.5 and 4.7% for PM10. While PM2.5 showed a modest effect in unadjusted models, its influence diminished markedly when adjusting for deprivation. These findings highlight the role of social and structural determinants, incorporating but reaching beyond direct pollutant exposure, in shaping respiratory health outcomes (O'Neill et al., 2003).

The modest association between PM2.5 and hospitalisations, compared with PM10, may reflect its smaller particle size and greater respiratory penetration (Schraufnagel et al., 2019a). The relatively low *R*^2^ values and non-significant interaction terms may reflect limited statistical power due to the modest sensor sample size and the overall high levels of deprivation across Liverpool wards (Higham et al., 2025; Acosta Ramírez & Higham, 2025; Public Health Institute, 2020). Comparable studies offer mixed evidence regarding the role of socioeconomic status as a modifier of pollution effects. In Rome, Forastiere et al. reported stronger PM10–mortality associations among lower socioeconomic groups (Forastiere et al., 2024), while Richardson et al. observed heightened susceptibility to PM10 in low-income European regions (Richardson et al., 2013). In the USA, education level appeared protective: Individuals with college education had a significantly lower risk of cardiorespiratory death from PM2.5 exposure than those with less than 12 years of education (do Nascimento & Gouveia, 2024). Other studies, however, found no significant modifying effects of education on pollution-related health outcomes (O'Neill et al., 2008; Ou et al., 2008). Our findings support the position that deprivation plays a consistent and independent role in shaping respiratory health risk. Differential health impacts of air pollution by deprivation level may result from both differential exposure (e.g. higher pollutant concentrations, indoor and outdoor) and differential susceptibility. This dual pathway is illustrated in our Directed Acyclic Graph (Fig. 5), which represents deprivation as both a potential confounder (when estimating pollution effects) and as an independent exposure mediated by air pollution. Additional factors, including indoor air quality and smoking, may compound these inequalities (Ferguson et al., 2020; Hiscock et al., 2012). Susceptibility may also be shaped by chronic stress, comorbidities, and reduced access to nutritious food and healthcare, which are all linked to deprivation (Barnett et al., 2012; Baum et al., 1999; Propper, 2024; The Food Foundation, 2022). Whilst we explored these pathways, independent or mediating effects of air pollution were not evident in our analysis, potentially due to relatively small sample sizes and/or geographical placement of monitors.

The reported air quality data from a 52-sensor network represents a significant development from the modelled data available in background maps and the single Automatic Urban and Rural Network (AURN) monitoring site for the Liverpool region, employed in previous analyses (Affairs, (Affairs n.d.); Dajnak & Beevers, 2020). With new sensors, calibrated by models and mobile sensors, the network provides an accurate view of Liverpool’s air quality, highlighting foci for targeted interventions to improve health and environmental standards. Liverpool presents a unique setting for this kind of analysis, combining multiple pollution sources, high deprivation, and excellent access to linked hospital and deprivation data. The availability of high-resolution sensor data, a composite measure of deprivation, and ward-level hospital data allowed for a wide-reaching and granular epidemiological assessment of environmental inequality. Potential limitations of this study include the use of geographically based rather than individual measures of deprivation and admission rates introducing a risk of ecological fallacy (Hajat et al., 2021). Whilst the 52-sensor network is a significant improvement on previously available data, this still involves a limitation to ward level analysis, which with a consequent aggregation of IMD data to the same level, limits the variability in this factor, and therefore the power of the analysis. Plans for additional stationary and mobile monitors will enable larger more powerful studies into the combined impact of pollution and other factors on population health in Liverpool. The use of emergency hospital admission for respiratory causes, whilst useful in view of its strong links with air pollution-related morbidity, could be critiqued for also incorporating elements of disease control, in the case of chronic conditions such as asthma. Chronic disease management is a complex variable, linked with deprivation levels through pathways mediated by air pollution (higher exposures triggering more exacerbations) as well as additional pollution-independent pathways (e.g., health literacy and access to healthcare) (Andrew Cumell, 2018). Additional analyses incorporating disease prevalence could add useful insights. Incorporation of factors such as ethnicity, also known to be important in these relationships, would also be informative (Busby et al., 2021).

Finally, whilst ambient air pollution data can indirectly reflect elements of indoor pollution such as exfiltration of pollutants from domestic combustion, it does not fully reflect individuals’ real exposures in their indoor environments (DEFRA, 2025; Ferguson et al., 2020). A further study drawing on indoor monitoring data is planned, which will provide more reliable information on these aspects of the pollution landscape. Building on these data, analyses of PM2.5 composition are underway, to reveal more about potential pollution sources and guide targeted interventions to improve health in the region. Detailed analysis of 2023–2024 monitoring data is planned, including time-series analysis of pollution-related admissions, to deepen understanding of contributing factors in Liverpool (Rushworth et al., 2014).

## Conclusions

This study draws on high-resolution data from a new 52-sensor air quality network to examine the associations between socioeconomic deprivation, particulate matter pollution, and respiratory health in Liverpool. Both PM2.5 and PM10 levels exceeded WHO guidelines (World Health Organization, 2021), with the highest concentrations observed in areas of greater deprivation and population density. Our analysis highlights the disproportionate burden of air pollution and deprivation on children’s respiratory health in the city. Deprivation emerged as a strong and consistent predictor of paediatric respiratory hospital admissions, explaining 16.1% of the variance, while PM2.5 and PM10 showed no statistically significant association with admissions when adjusted for deprivation, suggesting that deprivation acts as a key confounder in this relationship. This intersection reflects a clear environmental injustice: The communities least able to mitigate risk are those most exposed to harm (Jerrett et al., 2001). The sensor network offers a granular view of exposure across the city, identifying wards where health and environmental risks converge. However, the lack of statistically significant effects of PM in multi-predictor models underscores the need for larger datasets networks and additional variables to better capture the complex pathways at play. Future studies incorporating PM source attribution, time-series dynamics, and indoor exposures will provide deeper insights to guide interventions. Reducing paediatric respiratory morbidity in urban environments like Liverpool requires a dual approach: targeted reductions in particulate emissions through planning strategies (e.g., traffic controls, green infrastructure), and sustained investment in addressing the underlying drivers of deprivation and health inequality. Larger studies with enhanced statistical power are essential to confirm these patterns and support broader policy action to mitigate this inequitable burden.

## Data Availability

No datasets were generated or analysed during the current study.

## Declarations

All authors have read, understood, and complied as applicable with the statement on “Ethical responsibilities of Authors” as found in the Instructions for Authors.

## Clinical data

No clinical data is used.
